# Considerations in Platinum and Gold Drug Design and the Synthesis of Chloro(2,3-Diphenyl-1,3,4-Thiadiazolium-5-Thiolato-S_exo_)Gold(I): The First Gold Meso-Ionic Complex of Its Kind

**DOI:** 10.1155/MBD.1997.273

**Published:** 1997

**Authors:** B. P. Howe

**Affiliations:** Universidade Federal da Paraíba Departamento de Química CCEN Campus I João Pessoa Paraíba CEP 58.51-970 Brazil

## Abstract

It is evident that the chemistry of platinum is in a more advanced state than that of gold, mainly due to the success of the former in several anti-cancer drugs. With a view to finding possible, new candidates with chemotherapeutic potential, the use of sulphur-donor ligands bonded to platinum and gold is discussed herein in an attempt to promote the need to investigate similar ligands. Chloro(2,3-diphenyl-1,3,4-thiadiazolium-5-thiolato-S_exo_)gold(I) has been synthesised using a standard reaction, whereby Au(III) is initially reduced to Au(I) then reacted with the ligand, 2,3-diphenyl-1,3,4-thiadiazolium-5-thiolate.hydrochloride, isolated and finally characterised by elemental analyses and infrared spectroscopy. The compound is the first example of gold attached to a meso-ionic compound. It has also been tested for anti-bacterial and anti-fungal activity and shown to possess moderate activity against Gram-positive bacteria, although is inactive against, Gram-negative bacteria and fungi.


### CONSIDERATIONS IN PLATINUM AND GOLD DRUG DESIGN AND THE SYNTHESIS OF CHLORO(2,3-DIPHENYL-1,3,4-THIADIAZOLIUM-5-THIOLATO-$S_{exo}$)GOLD(I): THE FIRST GOLD MESO-IONIC COMPLEX OF ITS KIND

B. P. Howe

Universidade Federal da Paraíba, Departamento de Química, CCEN, Campus I, João Pessoa, Paraíba, Brazil, CEP 58.51-970. E-mail brianh@vm.npd.ufpb.br

### ABSTRACT

It is evident that the chemistry of platinum is in a more advanced state than that of gold, mainly due to the success of the former in several anti-cancer drugs. With a view to finding possible, new candidates with chemotherapeutic potential, the use of sulphur-donor ligands bonded to platinum and gold is discussed herein in an attempt to promote the need to investigate similar ligands. Chloro(2,3-diphenyl-1,3,4-thiadiazolium-5-thiolato-$S_{exo}$)gold(I) has been synthesised using a standard reaction, whereby Au(III) is initially reduced to Au(I) then reacted with the ligand, 2,3-diphenyl-1,3,4-thiadiazolium-5-thiolate.hydrochloride, isolated and finally characterised by elemental analyses and infrared spectroscopy. The compound is the first example of gold attached to a meso-ionic compound. It has also been tested for anti-bacterial and anti-fungal activity and shown to possess moderate activity against Gram-positive bacteria, although is inactive against Gram-negative bacteria and fungi.

### INTRODUCTION

Gold was reported as a healing agent as early as 2500 BC in the ninth province of China. Since then the application of gold has been extended mainly due to the implementation of chrysotherapy in the 1930's.[1a,1b] Gold compounds have been employed intensively in the search for new materials in various areas of medicinal chemistry, principally as anti-arthritics and anti-cancer drugs. Oxidation of gold drugs in the +1 state is considered improbable although there is experimental evidence to suggest that aurothiomalate, present in the commercialised gold sodium thiomalate (myocrisin), could be oxidised by myeloperoxidase or by hydrogen peroxide and hypochloric acid, which are products of burst polymorphonuclear leucocytes.[2]

In the period 1987-1994, most effort was directed at second-generation platinum(II) drugs containing diamino groups in the *cis*-position. Intrastrand DNA coupling was proved to be most prevalent as it is effected principally by *cis*-isomers.[3] On the other hand, a series of *trans*-platinum anti-tumour complexes were tested *in vitro* and *in vivo*. Surprisingly, of the complexes tested *in vitro*, many of them exhibited comparable potency to cisplatin whilst, in *in vivo* studies, all platinum (IV) complexes tested showed significant anti-tumour activity against the subcutaneous murine ADJ/PC 6 plasmacytoma model.[4] Clearly, the chemistry of platinum(IV) has still to be further developed.[5] Another interesting series of platinum(II) complexes bonded to acyclovir (a potent, metal-binding anti-viral drug) were synthesised and underwent biological testing. Unfortunately though, the anti-tumour activity of the Pt-acyclovir compex *cis*-$[PtCl(NH_3)_2(L)]NO_3$, where L = acyclovir i.e. 9-(2-hydroxyethoxymethyl)guanine, was markedly less than cisplatin when administered to P388 leukaemia-bearing mice.[6] Adducts of the anti-cancer drug carboplatin with sulphur-containing amino acids were investigated last year, as methionine and cysteine are thought to be responsible for the inactivation of Pt(II) complexes. The ring-opening of carboplatin and formation of relatively stable species was proven to influence the drug's biological activity.[7] Carboplatin reacts very slowly with thiols and this may be one of the reasons why it is less toxic than cisplatin. The bonds of any ligand to a platinum(II) centre ought to, in theory, be sufficiently resilient to ideally arrive at their site of action and be substituted by nitrogen bases of DNA, thus exerting their anti-tumour activity. High solubility within the body is also essential for the drug to be absorbed and assisted in reaching its target. Gold will react with molecules containing thiol residues present such as glutathione (a tripeptide), metallothionein and the Cys-34 link of albumin (proteins), amongst the many choices available, as they contain sulphhydryl groups; although the rates of such reactions have to be studied more fully.

*Gold and Platinum with Sulphur-Donor Ligands*

The *in vitro* and *in vivo* anti-tumour activity of gold complexes bound to thiolate ligands have been largely neglected. Thiol ligands attached to gold are easily replaced, making it very doubtful that a complex will arrive at its site of action without undergoing structural modifications. The synthesis of platinum complexes employing novel ligands similar to biomolecules is very popular and will no doubt be extended to gold. [8a-8f] 1,1-dithiolate compounds of transition metals were reviewed extensively by McCullough, although only a few sulphur-donors, e.g. dialkyldithiocarbamates, of platinum(II) and gold(I) were described.[9] Dithiolates of gold(I) with dianionic sulphur ligands bridging gold centres in di- and tri-nuclear complexes have also been reported *viz.*; 1,2-benzene dithiolate, (1,2-$S_2C_6H_4$); 1,3-benzene-dithiolate (1,3-$S_2C_6H_4$) and the 3,4-dimercaptotoluene analogue (3,4-$S_2C_6H_3Me$).[10] Metal compounds containing dithiolate ligands as illustrated in Figure 1 were also synthesised in the late 1980's and although platinum and gold derivatives were cited, no further work was performed on them.[11a,11b]

When $PtCl_4^{2-}$ is reacted with $S_4N_4$ (tetrasulphurtetranitride), complexes of platinum(II) of formula $Pt(S_2N_2H)_2$ or $Pt(S_3N)_2$ containing S,N bidentate ligands are formed. These are further examples of sulphur's ease in bonding to Pt, despite no mention being made to gold derivatives. Interestingly though, a product of formula $AuCl_2(S_3N)$ is obtained when $S_7NH$ is deprotonated with butyl lithium and added to chloroauric acid.[12a] The reaction in which tris(triphenylphosphine)platinum(0) forms a metallocycle is yet another example of a novel product:[12b]

$$Pt(PPh_3)_3 \quad + \quad S_4N_4H_4 \longrightarrow \quad Pt(NSNS)(PPh_3)_2$$

Furthermore, another compound $Pt(S_4N_3)Cl$, which contains the $S_4N_3^-$ group acting as a tridentate ligand bonding to platinum *via* two nitrogens and a suphur, is another fascinating example of the versatility of these ligands.[12c,12d]

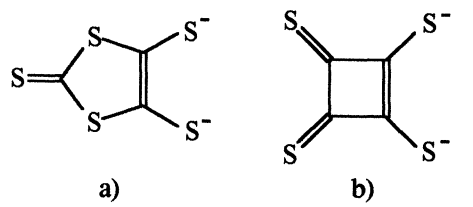

a) b)

**Figure 1. Dithiolate ligands a) 2-thiooxo-1,3-dithiole-4,5-dithiolate and b) tetrathiosquarate**

The use of bis(diphenylphosphine)methane sulphide yielded the first fully characterised gold methanide with P,S chelation to the precious metal in the example *cis*-$[Au^{III}(C_6F_5)_2(PPh_2CHPPh_2S)]$.[13] Housecroft's review of gold, which contains a section dedicated to sulphur-donor ligands, discusses new developments in gold(I) chemistry, highlighting some new complexes containing Au-thiouracil, -thioether and -mercaptooxopurines.[14] Finally, a vast number of meso-ionic compounds exist and are defined as planar five-membered heterocyclic betaines, possessing at least one side chain whose α-atom is also in the ring plane, and have a dipole moment of the order of 5D. To date, they have not been employed as ligands in either platinum or gold chemistry.[15] In spite of this, the compound 1,3-diphenyl-2-(4-chloro-3-nitrophenyl)-1,3,4-triazolium-5-thiolate and its hydrochloride were previously tested against three murine tumours: Ehrlich, Sarcoma 180 and B10MC11. The hydrochloride demonstrated anti-tumour activity *in vitro* and good efficacy against the Ehrlich tumour ($p<0.05$) when injected i.p.[16]

### MATERIALS AND METHODS

The meso-ionic compound, 2,3-diphenyl-1,3,4-thiadiazole-5-thiolate.hydrochloride was prepared by a literature method.[15] All reagents were purchased from the Aldrich Chemical Company and used without further purification, apart from $NaAuCl_4.2H_2O$ which was donated by Johnson-Matthey p.l.c.. Elemental analyses were performed on a Perkin-Elmer 2400 microanalyser (IQ-USP). Infrared spectra were recorded on a Bomem, Hartmann and Braun MB-Series spectrometer as potassium bromide discs in the range 4,000-400 $cm^{-1}$. Melting point determinations were performed on a Microquímica digital melting point apparatus, model MQAPF-301.

*Chloro(2,3-diphenyl-1,3,4-thiadiazolium-5-thiolato-$S_{exo}$)gold(I)*

Sodium tetrachloroaurate(III)(0.199 g, 0.5 mmol) was dissolved in water(30 ml) in a round-bottomed flask(100ml) and 2,2'-thiodiethanol(0.367 g, 3.0 mmol) syringed in with the gradual formation of a colourless solution of chloro(2,2'-dithioethanol)gold(I); the reaction being performed with cooling using ice under a nitrogen atmosphere. Immediately after addition of the meso-ionic compound(0.135 g, 0.5mmol) a bright yellow colour, slightly different to the ligand itself, was imparted to the colourless solution. The reaction was allowed to continue using magnetic agitation for 30 minutes. The product was then filtered off and redissolved in ethanol to finally yield shiny crystals which were dried in vacuo over phosphorus pentoxide. Yield 0.151 g (60%). F.W. 502.80. M.P. 150°C (dec). Elemental analysis $C_{14}H_{10}N_2S_2AuCl$: found(calculated) %C 33.39(33.44), %H 1.82(2.01), %N 5.27(5.57). I.R. (cm $^{-1}$) $\nu_{O-H}$ 3600-2800 (s); $\nu_{C=C}$ 1594 (mw), 1490 (m), 1455 (mw); $\nu_{C-N}$ 1397 (ms), 1184 (m), 1072 (s, w); $\delta_{C-H}$ in plane bending 1001 (w); $\delta_{C-H}$ out of plane bending 871 (m); $\delta_{C=C}$ in plane bending of monosubstituted benzene ring 760 (s); $\delta_{C=C}$ out of plane bending of monosubstituted benzene ring 682 (s); $\nu_{C-S}$ 570 (s); $\nu_{Au-S}$ 447 (w); $\nu_{Au-Cl}$ n.o.

*Biological Testing* [17, 18]

In the anti-bacterial testing, the medium used was Oxoid's Iso-Sentitest agar, whereas in the anti-fungal testing Oxoid's purified agar plus 10% yeast nitrogen base supplement was employed. A standard agar dilution was performed to determine the minimum inhibitory concentrations (MIC's) of the gold(I) meso-ionic compound. Stock solutions of the test compounds were prepared in dimethylacetamide. Varying aliquots of this or a further dilution (also dissolved in DMA) were added to a known volume of sterile media to give the following test range of compound: 100, 25, 10, 2.5, 1.0, 0.25 μg ml$^{-1}$ of agar. These were then poured into sterile 90 mm disposable Petri-dishes and allowed to solidify. The surface of these plates were inoculated with suspensions of test organism containing $10^4$ cells ml$^{-1}$. Plates were then incubated at 37°C for 24 h before being examined for their relative growth. MIC's were quoted as the range between the lowest concentration at which growth was observed for a particular organism and the highest concentration at which no growth was observed. Positive controls in each screen were ciprofloxacin (a quinolone anti-bacterial) and amphotericin B (a polyene anti-fungal drug). "Before" and "after" control plates were included; these were inoculated before and after test plates had been inoculated to ensure no interference due to carry-over of test compound. Control plates doubled as solvent controls, which themselves can be inhibitory at certain concentrations.

### RESULTS AND DISCUSSION

The reaction scheme described below is a general method for the preparation of gold(I) compounds. The ligand employed may be a phosphine, suphide or nitrogen-donor, although the donor strength of the ligand will vary depending upon the remainder of the molecule.[19]

$$AuCl_4^- \; + \; tdg \xrightarrow{0^oC} tdgCl_2 \; + \; AuCl(tdg) \; + \; Cl^-$$

$$AuCl(tdg) \xrightarrow{L} LAuCl$$

tdg = thiodiglycol = 2,2'-thiodiethanol

**Figure 2. Reaction scheme for the production of complexes of formula LAuCl**

A number of similar ligands containing a phenyl ring with different groups attached to them in positions 2, 3 or 4 would also produce new derivatives without interfering with the Au-S bond. The structure of chloro(1,2-diphenyl-1,3,4-thiadiazolium-5-thiolato-$S_{exo}$)gold(I) is represented below. Charge separation exists within the meso-ionic moiety as a partial positive charge is centred on the carbon atom in position-2 of the betaine, although spreads onto adjacent atoms $S_1$ and $N_3$. A corresponding negative charge is located mainly on the carbon atom in position-5 of the ring and extends towards the exocyclic sulphur atom as well as the adjacent $N_4$ atom. Hence, gold attaches readily to this sulphur atom through the one of its lone pairs, and also *via* the negative charge built up around $S_{exo}$ in preference to the sulphur atom in position-1 of the ring.[20]

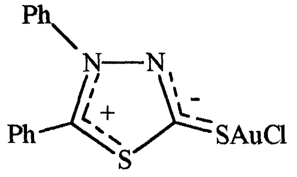

**Figure 3. The structure of chloro(2,3-diphenyl-1,3,4-thiadiazolium-5-thiolato-$S_{exo}$)gold(I)**

The results of biological testing against five different bacteria and four different fungi are displayed in Tables 1 and 2.

**Table 1. Anti-bacterial testing results for chloro(2,3-diphenyl-1,3,4-thiadiazolium-5-thiolato-$S_{exo}$)gold(I)**

| Bacteria | Minimum Inhibitory Concentration, MIC ($\mu g\ ml^{-1}$) | Ciproxfloxacin control |
|---|---|---|
| *Staphylococcus aureus* | 2.5 | <0.25 |
| *Enterococcus faecalis* | 2.5 | 1.0-2.5 |
| *Pseudomonas aeruginosa* | >100 | <0.25 |
| *Escherichia coli* | >100 | <0.25 |
| *Klebsiella pneumoniae* | >100 | <0.25 |

**Table 2. Anti-fungal testing results for chloro(2,3-diphenyl-1,3,4-thiadiazolium-5-thiolato-$S_{exo}$)gold(I)**

| Fungi | Minimum Inhibitory Concentration, MIC ($\mu g\ ml^{-1}$) | Amphotericin B control |
|---|---|---|
| *Candida albicans* | >100 | <0.25 |
| *Cryptococcus neformans* | >100 | <0.25 |
| *Aspergillus niger* | >100 | 0.25-1.0 |
| *Aspergillus fumigatus* | >100 | 0.25-1.0 |

The compound, chloro(2,3-diphenyl-1,3,4-thiadiazolium-5-thiolato-$S_{exo}$)gold(I), showed reasonably good activity against Gram-positive bacteria, although poor activity against Gram-negative bacteria was recorded; this being a common phenomena for metal complexes. Anti-fungal test results demonstrate that the compound is not significantly active, even though *Cryptococcus neoformans* is generally sensitive to metal compounds. As a final consideration, many gold meso-ionic compounds including substituted 1,3,4-triazolium-5-thiolates or 1,3,4-oxadiazolium-2-thiolates can be oxidised *via* halogens to their corresponding gold(III) derivatives and, thereupon, reacted with ligands which facilitate substitution of these groups. Ligands such as the silver salts of the anions displayed in Figure 1 will form part of our future investigations. Additionally, it is conceivable that gold(I) meso-ionic derivatives could react through substitutents in their aromatic ring systems and, for example, form poly-nuclear gold compounds.

### ACKNOWLEDGEMENTS

Financial support was kindly received via SERC (U.K.) and CNPq, a Brazilian government granting agency, in the elaboration of this work. The loan of the gold salt and biological testing via Johnson-Matthey p.l.c., Sonning Common, Reading, U.K., were very kindly appreciated.

### REFERENCES

1.
a. W.F. Kean, C.J.L. Lock, and H. Howard-Lock, *Inflammopharmacology*, **1**, 103, (1991)
b. G.G. Graham, G.D. Champion and J.B. Ziegler, *Inflammopharmacology*, **1**, 99, (1991)
2. M. Grootveld, D.R. Blake, T. Sahinoglu, A.W.D. Claxson, P. Mapp, C. Stevens, R.E. Allen and A. Furst, *Free Radical Res. Comm.*, **10**, 199, (1990)
3. V. Aletras, D. Hadjiliadis and N. Hadjiliadis, *Metal-Based Drugs*, **2.3**, 153, (1995)
4. L.R. Kelland, C.F.J. Barnard, I.G. Evans, B.A. Murrer, B.R.C. Theobald, S.B. Wyer, P.M. Goddard, M. Jones, M. Valenti, A. Bryant, P.M. Rogers and K.R. Harrap, *J. Med. Chem.*, **38**, 3016, (1995)
5. M. Galanski and B.K. Keppler, *Metal-Based Drugs*, **2.1**, 57, (1995)
6. M. Coluccia, A. Boccarelli, C. Cermelli, M. Portolani and G. Natile, *Metal-Based Drugs*, **2.5**, 249, (1995)
7. K.J. Barnham, M.I. Djuran, P. del S. Murdoch, J.D. Ranford and P.J. Sadler, *Inorg. Chem.*, **35**, 1065, (1996)
8.
a. A. Iakovidis and N. Hadjiliadis, *Coord. Chem. Revs.*, **135-136**, 17, (1994)
b. R.G. Buckley, A.M. Elsome, S.P. Fricker, G.R. Henderson, B.R.C. Theobald, R.V. Parish, B.P. Howe and L.R. Kelland, *J. Med.Chem.*, **39**, 5208, (1996)
c. G. Schröder, M. Sabat, I. Baxter, J. Kozelka and B. Lippert, *Inorg. Chem.*, **36**, 490, (1997)
d. E.G. Talman, W. Brüning, J. Reedjik, A.L. Spek and N. Veldman, *Inorg. Chem.*, **36**, 854, (1997)
e. E. Colacio, R. Cuesta, J.M. Gutiérrez-Zorilla, A. Luque, P. Román, T. Giraldi and M.R. Taylor, *Inorg. Chem.*, **35**, 4232, (1996)
f. K.F. Morris, L.E. Erickson, B.V. Panajotova, D.W. Jiang, F. Ding, *Inorg. Chem.*, **36**, 601, (1997)
9. R.P. Burns, F.P. McCullough and C.A. McAuliffe, *Advs. in Inorg. Chem. and Rad. Chem.*, **23**, 211, (1980)
10. M.C. Gimeno, P.G. Jones, A. Laguna, M. Laguna and R. Terroba, *Inorg. Chem.*, **33**, 3932, (1994)
11.
a. G. Matsubayashi, K. Akiba and T. Tanaka, *Inorg. Chem.*, **27**, 4744, (1988)
b. R. Grenz, F. Götzfriend, U. Nagel and W. Beck, *Chem. Ber.*, **119**, 1217, (1986)
12.
a. J.D. Woollins, *Polyhedron*, **3**, 1365, (1984)
b. P.F. Kelly and J.D. Woollins, *Polyhedron*, **5.3**, 607, (1986)
c. R. Jones, P.F. Kelly, D.J. Williams and J.D. Woollins, *Polyhedron*, **4**, 1947, (1985)
d. H. Endres and E. Galantai, *Angew. Chem., Int. Ed. Engl.*, **19**, 653, (1980)
13. A. Laguna and M. Laguna, *J. Organomet. Chem.*, **394**, 743, (1990)
14. C.E. Housecroft, *Coord. Revs.*, **127**, 187, (1993)
15. C.A. Ramsden, *Comp. Org. Chem.*, **4**, 1171, (1979)
16. T.O. Shinzato, N.F. Grynberg, R.M. Gomes, A. Echevarria and J. Miller, *Med. Sci. Res.*, **17**, 865, (1989)
17. S. Shadomy, *Appl. Microbiol.*, **17**, 871, (1969)
18. V. Lorian, Ed., *Antibiotics in Laboratory Medicine*, Williams and Wilkins, 2nd Edition
19. A.K.H. Al-Sa'ady, C.A. McAuliffe, R.V. Parish and J.A. Sandbank, *Inorg. Synth.*, **23**, 191, (1984)
20. K.-K. Cheung, A. Echevarria, S.E. Galembeck, M.A.M. Maciel, J. Miller, V.M. Rumjanek and A.M. Simas, *Acta Crystallographica*, **C49**, 1092, (1993)

**Received: August 8, 1997 - Accepted: September 8, 1997 - Received in revised camera-ready format: October 6, 1997**

